# Characteristics of the Gut Microbiota Composition of the Arctic Zone Residents in the Far Eastern Region

**DOI:** 10.3390/biomedicines12112472

**Published:** 2024-10-28

**Authors:** Alexandra I. Nekrasova, Irina G. Kalashnikova, Anna V. Korobeynikova, German A. Ashniev, Maria M. Bobrova, Sirozhdin Yu. Bakoev, Ekaterina S. Petryaikina, Alexander S. Nekrasov, Angelika V. Zagainova, Mariya V. Lukashina, Larisa R. Tolkacheva, Igor P. Bobrovnitskii, Vladimir S. Yudin, Anton A. Keskinov, Valentin V. Makarov, Sergey M. Yudin

**Affiliations:** 1 Federal State Budgetary Institution “Centre for Strategic Planning and Management of Biomedical Health Risks”, the Federal Medical and Biological Agency, Pogodinskaya Str., 10/1, 119121 Moscow, Russia; igkalashnikova@cspfmba.ru (I.G.K.); akorobeinikova@cspfmba.ru (A.V.K.); gashniev@cspfmba.ru (G.A.A.); mbobrova@cspfmba.ru (M.M.B.); sbakoev@cspfmba.ru (S.Y.B.); epetryakina@cspfmba.ru (E.S.P.); anekrasov@cspfmba.ru (A.S.N.); azagaynova@cspmz.ru (A.V.Z.); mlukashina@cspmz.ru (M.V.L.); ltolkacheva@cspfmba.ru (L.R.T.); vyudin@cspfmba.ru (V.S.Y.); keskinov@cspfmba.ru (A.A.K.); makarov@cspfmba.ru (V.V.M.); yudin@cspmz.ru (S.M.Y.); 2 Federal State Budgetary Scientific Institution “Institute of General Pathology and Pathophysiology”, Baltiyskaya Str., 8, 125315 Moscow, Russia; 1ipb@mail.ru; 3 State Scientific Center, the Russian Federation Institute of Biomedical Problems, the Russian Academy of Sciences, Khoroshevskoe Shosse, 76A, 123007 Moscow, Russia

**Keywords:** gut microbiota, microbiome, microbial diversity, adaptation, Arctic, cold stress

## Abstract

**Background**. In many studies over the past decade, scientists have made a connection between the composition of gut microbiota and human health. A number of publications have shown that gut bacteria are involved in many metabolic and physiological processes of the organism. The composition of the gut microbiome is unique for each person and is formed under the influence of various factors associated with both the individual characteristics of the body and the characteristics of the environment. Different regional characteristics make it necessary for the body to adapt to certain conditions, including temperature fluctuations. Living in areas with low temperatures, such as the Arctic zone, dictates the need for increased energy consumption, which affects the composition of the gut microbiome. **Methods.** In our study, an extensive questionnaire was conducted among the participants, where many questions were included about the dietary preferences of the study participants, which allowed them us to further divide them into groups according to their diets. Stool samples were collected from participants from 3 groups: Arctic native, Arctic newcomer and the control group. The next step was the isolation of bacterial DNA and sequencing the 16S rRNA gene. The analysis of the results of the diversity of the intestinal microbiota was carried out both with and without taking into account the dietary preferences of the participants. **Results**. As a result of comparing the intestinal microbiota obtained from residents of the Arctic zone with the gut microbiota of residents of other regions with a milder climate, significant differences are found. These differences may be related to limited food resources and a reduction in the variety of food products characteristic of this Arctic region. t was also found that representatives of the bacterial families *Christensenellaceae* and *Muribaculaceae* dominated the control group, both with traditional nutrition and with a dairy-free diet in comparison with the Arctic groups. The control group was dominated by representatives of the *Prevotellaceae, Enterobacteriaceae* and *Comamonadaceae* families compared to the Arctic group (with a traditional diet). The results also show that the number of representatives of the families *Desulfovibrionaceae* (with traditional diet) and *Enterobacteriaceae* (with milk-free diet) is growing in the Arctic group. **Conclusions.** In the course of this work, bacterial families characteristic of people living in the Arc-tic zone of the Far Eastern region of the Russian Federation were identified. Poor diet, difficult climatic conditions, and problems with logistics and medical care can have a strong impact on the health of this population. The main type of diet for the inhabitants of the Arctic is the traditional type of diet. They consume a large number of low-cost products, obtainget animal protein from poultry and canned food, and also eat a small number of fresh vegetables and fruits. Such a diet is due to the social status of the study participants and the climatic and geographical features of the region (difficulties in agriculture). With such a diet, we observe a decrease in representatives of the *Christensenellaceae, Muribaculaceae, Eubacteriaceae*, and *Prevotellaceae* families and an increase in representatives of the *Enterobacteriaceae* and *Desulfovibrionaceae* families among Arctic residents. This imbalance in the futuremay cause, this population may to develop various diseases in the future, including chronic diseases such as obesity, intestinal dysbiosis, inflammatory bowel diseases, and type 2 diabetes.

## 1. Introduction

The gut microbiota acts as another “organ” in the human body and participates in its functioning and metabolism [1]. The processes of human adaptation to changing environmental conditions went hand in hand with the coevolution of the human gut microbiome. During this process, mammals have developed various adaptive responses to environmental stressors [2], with gut microbiota playing a significant role in the host’s ability to adjust to changing conditions. Gut bacteria are involved in numerous physiological processes due to their metabolic activities. Therefore, researchers have identified cross-axes of interaction between the intestine and other body systems, including the immune, nervous, and cardiovascular systems [3]. The composition of the gut microbiome is unique to each individual and is shaped and modulated by a range of factors, including climatic and geographical environmental conditions [4]. Seasonal temperature fluctuations, living in northern regions, and the development of extreme habitats all serve as prerequisites for the adaptation of both the host organism and its microbiome to cold stress and the limited food diversity typical of these environments.

The territories of some countries are located in regions beyond the Arctic Circle (https://www.arcticcircle.org/, accessed on of 1 September 2021) or in close proximity to it. It is known that, in this geographical area, the body of people who arrived from regions that are more southern finds itself in an environment characterized by extremely unfavorable natural and climatic conditions, which undoubtedly has a significant impact on the physiology of the body [5]. This issue is especially relevant for the Russian Federation, where regions exhibit significant variability in relief and climate. A substantial portion of the territory consists of Arctic regions, the Far North, or areas classified similar to those of the Far North. The influence of numerous stress agents common in the Arctic regions—such as the disruption of the daily routine associated with such a phenomenon as the polar night [6], cold, poor environmental conditions due to the extraction of heavy metals and minerals, poor diet, and exposure to toxic elements and organic substances—negatively affects the state of the intestinal microbiome [5,7].

In our research, we aimed to investigate the composition of the intestinal microbiota in both indigenous and non-native populations residing in the Arctic zone. Our goal was to identify the bacterial families associated with the climatic conditions and dietary habits of these groups, as well as to identify potentially adaptogenic groups of intestinal bacteria.

Studies dedicated to the impact of geographical factors on the intestinal microbiota composition have revealed a correlation between the region of residence and dietary habits with the variability of specific microbiome taxa. The bacterial composition of the gut is interlinked with the region of human residence due to territorial isolation and climatic, nutritional, and cultural characteristics, which also serves as evidence of the fact that the gut microbiota undergoes changes depending on environmental factors and participates in the adaptation to certain environmental conditions. Many researchers have noted certain differences in the composition of the gut microbiota depending on the region of residence. For example, it has been shown that there are more representatives of the *Bacteroides* genus in the intestinal microbiota of Chinese residents than in European residents, which may be due to differences in lifestyle [8]. The influence of the geographical factor on the gut microbiota in the European population has also been well studied. An analysis of the fecal microbiota of children in six clinical centers in Europe (Finland, Sweden, and Germany) and the United States (Colorado, Washington, Georgia, and Florida) showed a significant association of geographical origin with the abundance and diversity of various bacterial genera, especially *Bifidobacterium*, *Veillonella*, *Faecalibacterium*, *Streptococcus,* and *Akkermansia* in the gut microbiota [9]. The study revealed significantly lower gut microbiota diversity in infants from Finland and Colorado, which is consistent with an earlier report on a decrease in gut microbiota diversity in infants from Northern Europe compared to infants from Southern or Central Europe, regardless of the method of childbirth, breastfeeding, and antibiotic effects [9]. The results of this research indicated that the children from the northern regions had a higher proportion of *Bifidobacterium*, while more diverse microbiota with a larger number of *Bacteroidaceae* representatives was typical for those in the southern countries [10].

However, information about the microbiota characteristics of residents from extremely cold regions is limited and often contradictory. One of the research studies analyzed the differences in the gut microbiota of Canadian Inuit living in the Arctic zone with the microbiota of southern Canadians and European groups [11]. The authors observed that the Inuit possess a variety of intestinal bacteria, if compared with urbanized American and Western populations, largely due to dietary changes (specifically, the shift from a traditional to a Western diet). However, notable differences in the gut microbiome composition between the Inuit and European populations have been identified, potentially attributable to both genetic and geographical factors. For instance, variations in the abundance and strains of *Prevotella* among these groups have been identified [11].

It is worth noting that geographical features and climatic conditions are most often associated with the food preferences of the resident population. For example, a study of European children (following a Western-type diet) and children from rural Africa (whose diet is rich in millet, sorghum, and local vegetables, characterized by a low content of animal proteins and lipids of animal origin) showed that the microbiota of African children is characterized by an elevated abundance of *Prevotella* and *Xylanibacter* bacteria, which are involved in the hydrolysis of cellulose. In addition, *Enterobacteriaceae* (*Shigella* and *Escherichia*) were less represented in the microbiota of African children versus European children [12].

Recent research has revealed a correlation between the high incidence of colorectal cancer among Alaskans and the composition of their gut microbiota. A population of South African residents from the coastal region, characterized by a low risk of developing colorectal cancer, was selected as a comparison group [13]. The families *Ruminococcaceae* and *Prevotellaceae*, covering the main genera involved in saccharolytic fermentation, have shown lower abundance in the microbiota of the Alaskan residents. Within the other most common genus, their gut microbiota demonstrated a substantially higher number of microorganisms belonging to the *Bifidobacterium*, *Escherichia,* and *Shigella* genera. The gut microbiota of South Africans had a significantly higher number of bacteria associated with the *Prevotella*, *Succinivibrio*, and *Ruminococcaceae* families and *Eubacterium coprostanoligenes* [14]. The microbiome features were explained by the different diets of the participants. The diet of Alaskans is characterized by significantly higher levels of animal protein and retinol, reflecting a nutritional profile rich in meat, fish, and seafood. In contrast, the diet of Africans included a considerably greater intake of total carbohydrates, dietary fiber, and vegetable protein. Additionally, South Africans exhibit low retinol consumption, which contributes to the vitamin A deficiency within the population [14,15].

A study examining the gut metagenome of healthy residents in Russia revealed differences in microbiota composition among residents from various climatogeographic regions [16]. The residents of rural Siberian regions had a predominance of *Prevotella*, *Faecalibacterium*, and *Coprococcus* genera and *Lachnospiraceae* family if compared with residents of middle latitudes. The authors indicate that the differences in microbiota composition among residents from various regions of the country are associated with variations in their living conditions. Additionally, other large-scale studies have demonstrated that the regional characteristics of host microbiota are closely linked to abundance fluctuations in microbiota [17].

Based on the data presented, it can be concluded that geographical factors, including the place of residence, environmental conditions, and dietary characteristics, are directly linked to microbial diversity and play a crucial role in shaping the gut microbiota. Dietary habits and climatic conditions are important determinants of the regional gut microbiota characteristics. A noticeable trend suggests that, in colder regions, there is a shift in microbiota composition, due to increased caloric intake, and characterized by a higher abundance of *Bacteroidetes* and a lower abundance of *Firmicutes*.

## 2. Materials and Methods

### 2.1. Research Design and Participants

The research involved 98 indigenous Arctic residents (including Yakuts, Evenks, Nenets, Khanty, and others) and 59 newcomers who had lived in the Far North for a duration of 1 to 3 years. A control group consisted of 51 residents from the central region of the Russian Federation.

The main limitation in the size of the sample of native and newcomer groups is the low population density in these areas (the population of Tiksi village in 2021 is 4173 people, and that of the village of Chokurdakh is 1869 people). The low population density is associated with harsh climate conditions (low temperatures and polar night) and the low development of these settlements in the Far North.

The study participants of all groups represent a population with a large ethnic diversity, as a result of which it can be called a mixed population based on ethnicity. The following criteria were selected for the study, as inclusion and non-inclusion for donors: Inclusion criteria:

- Male gender;
- Aged from 18 to 50 years;
- Belonging to health groups 1 or 2 (conditionally healthy people);
- Voluntary informed consent to participate in the study and all types of testing provided for by the study.

Criteria for non-inclusion:

- The presence of severe clinically significant neurological, mental, cardiovascular, endocrine, gastrointestinal diseases, liver and urinary system diseases, immune diseases in the anamnesis;
- Taking antibacterial, antihistamine, and/or narcotic drugs for two weeks before the studies;
- The presence of alcohol dependence/drug addiction/substance abuse; or taking pro-/prebiotic medications a week before participating in the research;
- The presence of signs of ARVI during examinations;
- Refusal to fill out the questionnaire and/or sign an informed consent form to participate in the study and all testing methods provided by the study.

The participants in this study belonged to the middle socio-economic class. Most of the participants had secondary education and had graduated from college. The income of this group was considered to be at a middle level, with an average monthly salary of about USD 900.

The majority of the participants can be described as blue-collar, with the most common professions being worker, boiler room operator, driver, technician, locksmith, and security guard. Some individuals were engaged in reindeer husbandry, hunting, and fishing. The newcomer population primarily worked on a rotational basis, as they came to the Far North region for fixed periods of time due to the higher wage levels.

All participants filled out special forms that included general information about the donor, nutrition information, and medical history. The interview was conducted under the control of a medical specialist. The average value of the body mass index (BMI) for all groups contains the range of normal weight (BMI 25–29.9—normal weight) (Table 1).

All participants took a questionnaire, where they were asked in detail about their food preferences, the presence of chronic diseases, taking medications, etc. The developed form of the standardized questionnaire is given in Supplementary Materials, Microbiota Donor Questionnaire.

### 2.2. Collection, Storage, and Transportation of Samples

The inclusion of the study participants took place from the end of August to the beginning of November 2021.

The collection of fecal samples from the native and newcomer populations of the Russian Arctic and the delivery of biological samples to the site for further research were carried out in accordance with the research protocol. The samples were collected in the territory of Allaikhovsky and Bulunsky uluses of the Republic of Sakha (Yakutia) and the Omsk region during field trips, medical centers of Tiksi, Chokurdy, and Omsk hospital. Fecal samples were taken from 208 conditionally healthy (health groups 1 and 2) men of working age after receiving written voluntary informed consent from them.

The selection was made under normal conditions using sterile containers on the day of the examination. The samples were delivered to the receiving point no later than 2–4 h after defecation. The storage and transportation of the biological samples was conducted at a temperature of −80 °C without defrosting. The samples were transported in a thermobox with dry ice.

### 2.3. Ethical Provision of Research

Before the start of the scientific work, a form of informed consent of the donor was developed to provide biological samples for research, as well as to conduct a questionnaire. The development of a form of informed consent of the donor for the provision of biological samples and conducting a questionnaire was carried out on the basis of Appendix No. 2 to the Order of the Ministry of Health of the Russian Federation dated 20 December 2012 No. 1177n (as amended. Dated 10 August 2015) “On approval of the procedure for giving informed voluntary consent to medical intervention and refusal of medical intervention in relation to Certain Types of medical interventions, forms of informed voluntary consent to medical intervention and forms of refusal of medical intervention” and in accordance with the Federal Law of 21 November 2011. No. 323-FZ “On the basics of protecting the health of citizens in the Russian Federation” and the Helsinki Declaration of the World Medical Association “Recommendations for doctors on conducting biomedical research with human participation as an object of research”.

Research was conducted in accordance with the ethical principles of medical research with human participation (Helsinki Declaration of the World Medical Association, 1964–2013). The Ethics Committee of the RUDN (Peoples’ Friendship University of Russia) Institute (Protocol No. 30 dated 17 June 2021) approved the research protocol, the text of the information sheet, and the informed consent of the patient.

### 2.4. Metagenomic Analysis of Microbiota Specimens

Bacterial DNA was isolated from fecal samples using a QIAamp DNA Stool Mini Kit (Qiagen, Hilden, Germany), according to the manufacturer’s instructions. The concentration and quality of the isolated DNA were measured with a Qubit 4 fluorimeter (Thermo Scientific, Waltham, MA, USA), and the size of the obtained bacterial DNA was determined using an Agilent D100 reagent kit on an Agilent 4200 TapeStation device (Agilent Technologies, Inc., Santa Clara, CA, USA).

Metagenomic analysis of intestinal microbiota samples was performed using the high-throughput sequencing (NGS) method by targeted sequencing and subsequent bioinformatic analysis of the V3–V4 nucleotide sequence of the 16S ribosomal RNA gene site on the Illumina MiSeq sequencer (Illumina, San Diego, CA, USA). The concentration and quality of libraries were measured using a Qubit 4 fluorimeter (Thermo Scientific, USA). The size of the resulting libraries was determined using the Agilent D1000 reagent kit on the Agilent 4200 TapeStation device (Agilent Technologies, Inc., USA).

### 2.5. Bioinformatic Data Processing

The metagenomic analysis was conducted on samples of gut microbiota received from the newcomer and the native population of the Arctic zone of the Russian Federation. The analysis included 279 samples in the form of paired-end (2 × 300 nucleotides) reads of the Illumina MiSeq V3–V4 region of the 16S rRNA gene with a median number of reads of 175,000 per sample. Then, the sequencing quality was evaluated using FastQC and MultiQC, during which we determined the filtering and trimming parameters of the obtained 16S rRNA reads. Subsequently, we implemented the QIIME2 pipeline. QIIME2 (Quantitative Insights Into Microbial Ecology 2) is a powerful tool for analyzing the composition and diversity of microbial communities based on 16S rRNA sequencing data.

The following are the primary steps of data processing:

1. Import sequencing data;
2. Reads filtering;
3. Combining paired-end reads;
4. Alignment of the obtained ASVs and construction of a phylogenetic tree;
5. Selection of representative ASV sequences;
6. Establishing a taxonomy using naive Bayesian classifiers;
7. Analysis of alpha and beta diversity. Visualization of results using Python (v. 3.10) using a jupyter notebook (v. 7.2.0), R (v. 4.2.3) using RStudio (v. 2022.02.2, Build 485), and emperor plot;
8. Statistical processing of the received data.

The list of families identified during the analysis is presented in Supplementary Material, Table S1.

### 2.6. Data Preprocessing

The work uses data from classifiers for the RDP and SILVA databases, which were used in parallel to obtain reliable results. The main difference between RDP and SILVA is their coverage. RDP describes the taxonomic affiliation of ribosome RNA sequences of bacteria and archaea, while SILVA covers all domains of life, including bacteria, archaea, and eukaryotes. At the same time, RDP is a supervised database with the most accurate taxonomic assignments, whereas SILVA includes sets of ribosome RNA sequences that are rare and uncultivated.

The benefit of the RDP database is more accurate quantitative data, while SILVA has more extensive species representation. In addition to the quantitative data, questionnaire materials containing information about the belonging of people to three groups were involved, as follows: newcomer (resident of the Arctic), native (resident of the Arctic), and control (persons living in the central part of Russia). First, each group was considered separately, then the data on newcomers and natives were combined into the “Arctic” group and considered together in respect of the control group.

For analysis, all files were converted to the phyloseq (4S) format, which stores related information about taxonomy, metadata, and quantitative calculations (reduced to the generally accepted ASV format). The transformation was performed using the phyloseq package (v. 1.42.0), and the final object contained 4 linked files, including samples metadata, taxonomy, ASV table, and taxonomic tree.

The preprocessing stage included several stages. At the beginning, the bacteria were combined to the level of families with the application of custom normalization to low-represented taxa, as follows:

- If the number of accounts is <31, take it as 0;
- Mark all values 31–99 for 75;
- Values in the range 100–149 should be designated as 125;
- Values outside of the specified ranges remained unchanged.

Families identified as unidentified (“NA”, “uncultured”, “__”), as well as those containing exclusively zero values, were removed from further analysis. In the final version, the classification data for the RDP and SILVA databases contained information on 55 and 66 families, respectively.

For statistical analysis, an alpha diversity table with the following six indicators of biodiversity was used: Shannon entropy (shannon_entropy), phylogenetic diversity (faith_pd), pielou_evenness index, abundance index (chao1), Simpson diversity index, and strong dominance index.

### 2.7. Methods of Microbiota Data Analysis and Statistical Processing

#### 2.7.1. Multivariate Test for Differences in Total Composition Between Groups of Samples

This stage includes a primary general assessment of the groups, comparing them in pairs with each other. This test is based on calculating the differences between the average distributions of each taxon, taking into account the excessive spread of the counting data. The work was carried out using the HMP package (v. 2.0.1). The methodology described in the article served as the basis for the analysis (https://journals.plos.org/plosone/article?id=10.1371/journal.pone.0052078, accessed on 13 May 2024).

#### 2.7.2. Alpha Diversity

For each sample we calculated the following 6 indices of alpha diversity:

(1) Shannon entropy measures both species richness and evenness within a sample. This index is sensitive to the presence of rare species.
(2) Faith index measures the total branch length of a phylogenetic tree that connects all of the species present in a sample and is based on summing the branch lengths that connect the observed taxa.
(3) Pielou evenness index measures how evenly community representatives are distributed across the different species. It is calculated by Shannon entropy normalization as a logarithm of the observed species richness.
(4) Chao1 measures species richness by considering the number of observed species and the frequency of rare species (singletons and doubletons).
(5) Simpson diversity index measures the probability that two randomly selected community representatives belong to the same species.
(6) Strong index focuses on the most dominant species within a community, providing a measure of how much a single species dominates the community composition.

To ensure accurate calculations of these indices, the input feature table was filtered and rarefied to a sampling depth of 20,000 in order to minimize biases and reflect the true diversity of the gut microbiota samples.

For the evaluation of groups based on six available indicators, scale diagrams were constructed for three groups, a pairwise comparison of samples for each taxon was performed using the Mann–Whitney criterion, and a general statistical estimate for three groups was obtained using the Kolmogorov–Smirnov test. The calculated *p*-values were adjusted for FDR to minimize false positive results. The calculations were performed with the rstatix package (v. 0.7.2) and visualization was performed with the ggpubr package (v. 0.6.0).

#### 2.7.3. Beta Diversity

Beta diversity provides a measure of the similarity or disparity of one microbial composition with another. It is computed directly from the ASV table after normalization. In our example, the microbiome data were converted according to the formula log10(x). The paper uses the calculation of the Unifrac distance (weighted and unweighted metrics), a method for measuring beta diversity that uses phylogenetic information to compare groups. The analysis was performed using the ordinate() function of the phyloseq package (v. 1.42.0) using the PCoA method (distances “wunifrac” and “unifrac”). The reliability of the differences in beta diversity between the groups was calculated by the permutation multivariate analysis of variance test, which is implemented in the adonis2() function of the vegan (v. 2.6-4) package.

#### 2.7.4. Differential Population Analysis

Differential abundance analysis is one of the main methods used to find differences in samples in the microbial community and identify taxa associated with certain environmental, biological, or clinical factors. Nonparametric tests are preferable because microbiome data do not have a normal distribution when tested by the Shapiro–Wilk method. The goal of this step is to identify specific taxa of interest in terms of changes in the composition of the microbiota in people with different living conditions. A nonparametric Mann–Whitney test was performed, and the *p*-value was calculated and adjusted for FDR to minimize false positive results. The calculations were performed with the rstatix package (v. 0.7.2).

#### 2.7.5. Linear Discriminant Analysis Effect Size (LEfSe)

The available R LEfSe (linear discriminant analysis effect size) package was used to perform linear discriminant analysis with phyloseq format data (LEfSe package v. 1.8.0). The effect of linear discriminant analysis is a convenient method for identifying genomic biomarkers to characterize statistical differences between biological groups.

#### 2.7.6. Machine Learning Models

The Random Forest machine learning model was used to build binary classification models. The quality of the model was assessed by the OOB (out-of-bag error) metric, which makes it possible not to divide the sample into a training sample and a test one. The stage was executed using the RandomForest package (v. 4.7-1.1) and paralleled using the doParallel library (v. 1.0.17).

#### 2.7.7. Correlation Analysis

Correlation analysis is a statistical method that can show the correlation of variables. The corrplot package (v. 0.92) was used to realize this stage. A *p*-value score (psych package v. 2.4.1) was obtained for each pair. The *p*-value is calculated based on the number of pairs, as follows: if there are few pairs, then the correlation coefficient should be very close to the peak values; if there are many, then a small coefficient can still be considered significant, which is especially useful if there are many zeros in the input matrices.

#### 2.7.8. Analyzing the Effect of Diet

The groups were divided into the following four types of diets: traditional, healthy, milk-free, and sugar-heavy diets. The Mann–Whitney statistical test was performed similarly to that outlined in Section 2.7.2 and Section 2.7.4, but without adjustment because of the small statistical power of the groups. Visualization was performed with the ComplexHeatmap package (v. 2.20.0) using min–max normalization.

## 3. Results

### 3.1. Multivariate Test for Differences in Total Composition Between Groups of Samples

According to the results of the HMP test (Table 2), we reject the zero hypothesis that there are no differences in the distribution of the bacterial family counts between the groups, since the *p*-value values turned out to be below the significance threshold (α = 0.05).

### 3.2. Alpha Diversity Analysis

**Figure 1:**
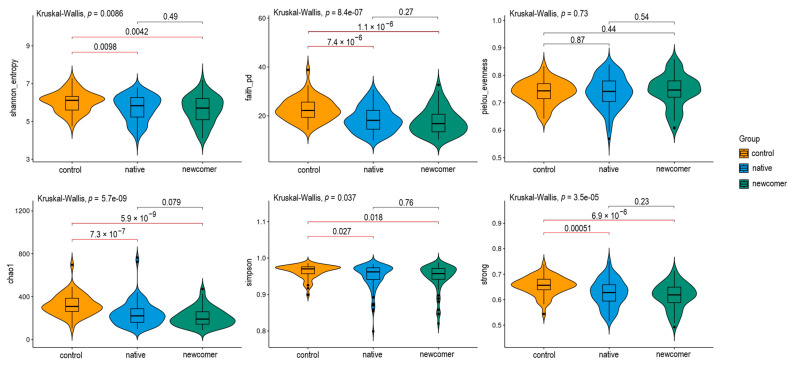
Alpha diversity testing results.

A nonparametric statistical Mann–Whitney test was performed for alpha diversity data. Figure 1 shows the *p*-value results above each line. Indicators with a significant *p*-value are marked with red lines (the accepted threshold is α = 0.05).

**Figure 2:**
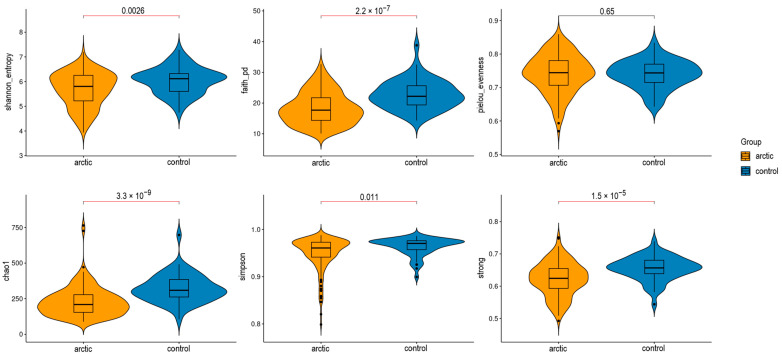
Alpha diversity testing results for Arctic//control groups.

The overall assessment of the significance of each presented indicator was conducted using the Kruskal–Wallis criterion. According to the test results, the threshold of significance (α = 0.05) was not reached only for the Pielou equalization index (pielou_evenness), which takes into account the proportion of each species relative to the total number of all species. This percentage is utilized to determine the uniformity index, which measures how evenly species are distributed within a community. When combined with other diversity metrics, such as the Simpson Index or the Shannon Index, the Pielou uniformity index provides a more comprehensive description of the community structure. The calculated value ranges from 0 (indicating a lack of uniformity) to 1 (indicating complete uniformity). The newcomers-control and native-control groups showed significant results when analyzed with the Mann–Whitney test for all indices that exceeded the relevance threshold. Furthermore, the newcomer and native population groups were combined into an “Arctic” group and compared to the control group (samples from the central region of Russia). The results were similar to those previously presented across three groups and are displayed in the figure below (Figure 2).

### 3.3. Beta Diversity Analysis

**Figure 3:**
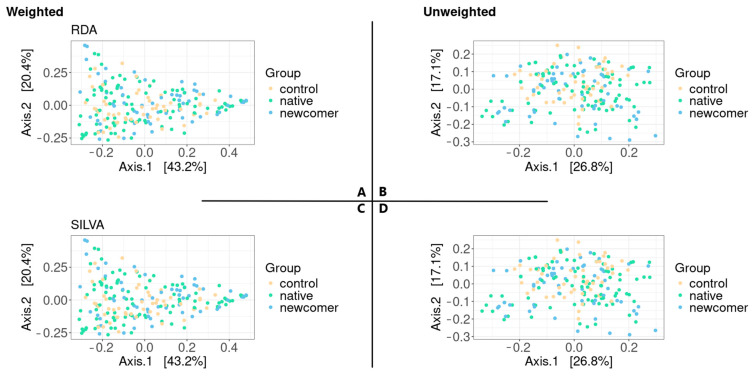
Beta diversity test results. (**A**,**C**)—weighted UniFrac according to RDP and SILVA, respectively; (**B**,**D**)—unweighted UniFrac according to RDP and SILVA, respectively.

Phylogenetic tree information was introduced into the beta diversity analysis. The UniFrac metric includes phylogenetic information by calculating the total length of branches “undivided” between two samples divided by the total length of the branches. The approach is capable of identifying differences in phylogenetic affinities between specimens and specimen types. Here, we calculate the weighted and unweighted UniFrac metrics using PCoA (Figure 3).

The clustering of samples by groups is not obvious based on ordination graphs. We conducted a variance analysis using distance matrices to obtain an estimate of the statistical significance of the differences between the groups (Table 3). Therefore, the result turned out to be statistically insignificant only in the case of unweighted UniFrac in RDP.

### 3.4. Differential Population Testing

A nonparametric Mann–Whitney test was performed, and the *p*-value was calculated and adjusted for FDR to minimize false positive results. The LEfSe tool is also involved in the analysis, which identifies the features that are more probable to explain the differences between the classes by combining standard tests for statistical significance with additional tests encoding biological consistency and relevance of effects. Class comparison methods usually predict biomarkers consisting of features that violate the zero hypothesis of the absence of differences between classes. The run LEfSe() function was started with default parameters, and the results are shown in the tables below (Table 4 for RDP and Table 5 for SILVA).

The families *Eubacteriaceae* and *Christensenellaceae* demonstrate a significant difference in all tests of the control group, the *Enterobacteriaceae* family also demonstrates significance in almost all of the control tests, except for the LEfSe analysis of the control-newcomers groups.

The *Christensenellaceae* and *Enterobacteriaceae* families demonstrated significant results when comparing the control group in all variants.

### 3.5. Machine Learning Models Analysis

All comparison groups with the control sample demonstrated high classification accuracy while constructing the Random Forest model (Table 6). The comparison of the newcomer and native groups had a far too high a percentage of errors, and, for this reason, the results could not be considered reliable and were not taken into account further in the analysis.

**Figure 4:**
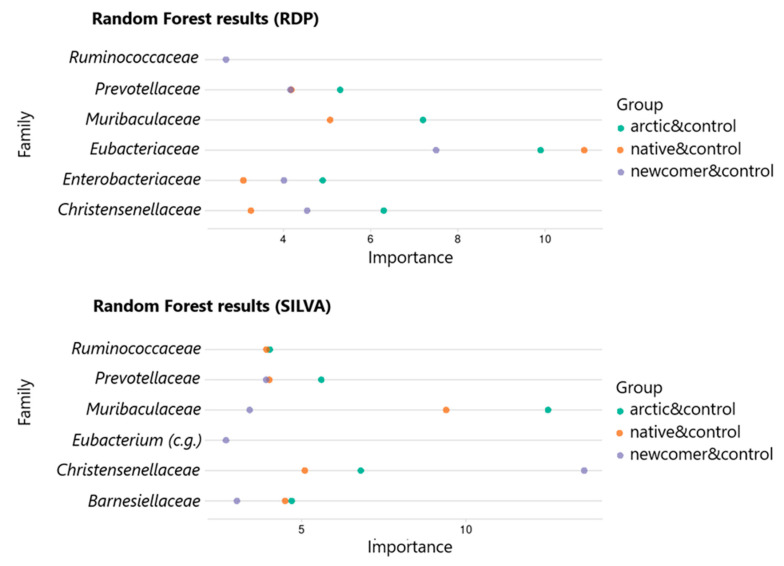
Results of the RF model (SILVA).

A list of the five most important bacterial families was obtained from each model using “Gini importance” metric ranking. The result for all groups is reduced to the form of a common table and visualized using a ggplot2 package (Figure 4).

In the case of the RDP classifier, the *Eubacteriaceae* family demonstrated the greatest significance for all comparison groups, and the *Ruminococcaceae* family turned out to be significant only when comparing the newcomer population and the control group. In the case of the SILVA classifiers, the *Christensenellaceae* family demonstrates the highest assessment of significance when comparing the newcomer population and the control group and the *Muribaculaceae* family for the other two comparison groups. The *Eubacterium coprostanoligenes* group (c.g.) is also significant only when considering the “newcomers and control” groups.

### 3.6. Results of Correlation Analysis

**Figure 5:**
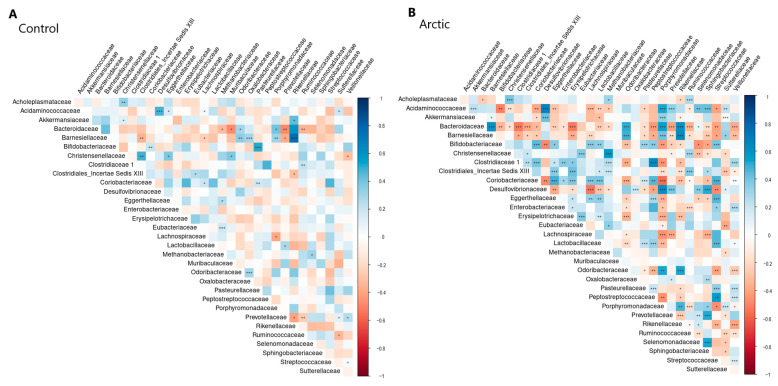
Correlation analysis results (RDP). Significance is denoted as follows: <0.001: ‘***’, <0.01: ‘**’, <0.05: ‘*’.

**Figure 6:**
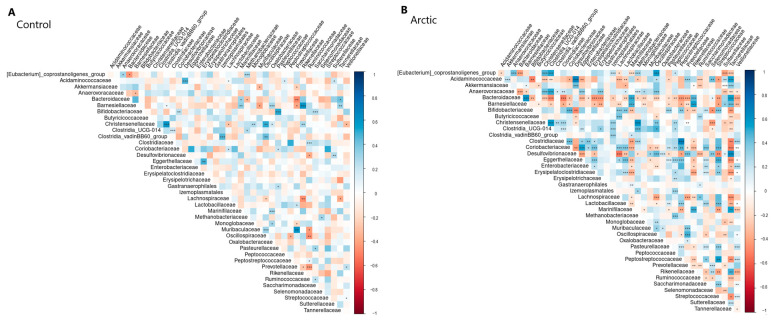
Correlation analysis results (SILVA). Significance is denoted as follows: <0.001: ‘***’, <0.01: ‘**’, <0.05: ‘*’.

Correlations between all bacteria were calculated within the Arctic and control groups, and the *p*-value for each pair was obtained (with clarification by FDR). The results are visualized in Figure 5 for RDP and in Figure 6 for SILVA. All statistically significant correlation pairs (α = 0.05) were selected, in which the nature of the relationship between the analyzed groups changes (positive correlation changes to negative and vice versa). Couples in which there was a change (strengthening or weakening) in the bond were also identified. Table 7 shows the results.

Based on the results obtained, a summary table has been compiled, which includes only those bacterial families that were detected by all three methods used (Mann–Whitney testing, LEfSe, and Random Forest).

Table 8 shows that the composition of the gut microbiota of the newcomer and native populations living in the Arctic zone significantly differs from the composition of the gut microbiota of the control group. It was observed that the diversity of microbial composition decreases and the number of representatives of the families *Christensenellaceae*, *Prevotellaceae*, *Muribaculaceae,* and *Eubacteriaceae* decreases among the residents of the Arctic zone. At the same time, the Arctic residents have an increased number of representatives of the *Enterobacteriaceae* and *Selenomonadaceae* families in comparison with the control group. The residents of the newcomer group demonstrated an increase in representatives of the *Acidaminococcaceae* family in comparison with the native group and the control and in the newcomer population, and there was an increase in representatives of the *Desulfovibrionaceae* family in comparison with the control group. Thus, it could be observed that the groups of native and newcomer populations living in the Arctic zone have a decreasing number of representatives of the intestinal microbiota families that bind the microbiota of a conditionally healthy person (for example, *Christensenellaceae* and *Muribaculaceae*).

The next step was to compare the bacterial diversity and the number of representatives of bacterial families between the Arctic and control groups, taking into account food preferences.

Questionnaires describing the food preferences of the study participants were analyzed and divided into groups typical of people living in the territory of the Russian Federation. The following four dietary groups were identified: a traditional diet (traditional), a milk-free diet (milk-free), a diet with a high sugar content (sugar-heavy), and a healthy diet (healthy). The traditional diet is characterized by a high content of complex starchy carbohydrates (represented by cereals, pasta, and potatoes), animal protein (mainly poultry meat), and this diet is also rich in ultra-processed foods (sausages, salami, and canned food), as well as a low content of fresh vegetables and fruits. The milk-free diet is characterized by the exclusion of whole cow’s milk and cream from the diet. The sugar-heavy diet is characterized by an increased content of simple carbohydrates (bakery and confectionery products, chocolate, sugary drinks, etc.). The healthy diet is characterized by the high consumption of fresh vegetables and fruits, animal protein in the form of poultry and fish meat, fermented dairy products (yogurt, kefir, cottage cheese, etc.), and the low consumption of simple carbohydrates (bakery and confectionery products, chocolate, sweet drinks, etc.), and ultra-processed foods (sausages, fast food, and junk food).

Because these samples by diet are not balanced and limited in number, we cannot consider our result reliably robust. Nevertheless, we consider possible patterns in the variation of the content of different bacterial families depending on the type of diets.

**Figure 7:**
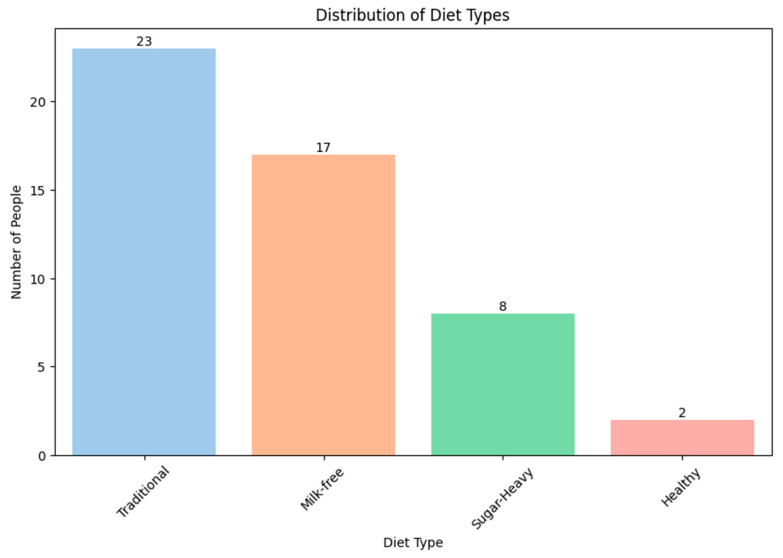
The distribution of participants into groups according to food preferences (Arctic group).

**Figure 8:**
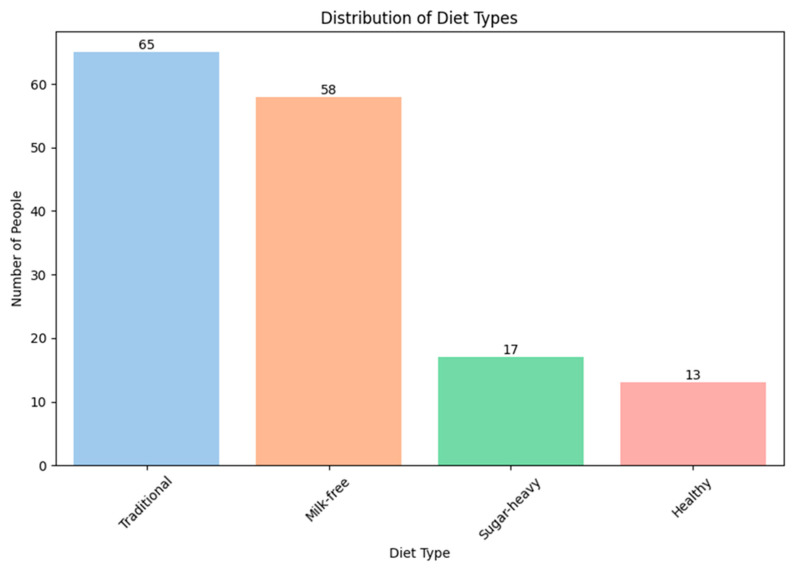
The distribution of participants into groups according to food preferences (control group).

In the Arctic and control groups, the participants that adhere to a traditional diet are most common (Figure 7 and Figure 8). This is due to limited resources related to the geographical features of the area and difficulties with the logic of fresh food, as well as the social status of the participants.

**Figure 9:**
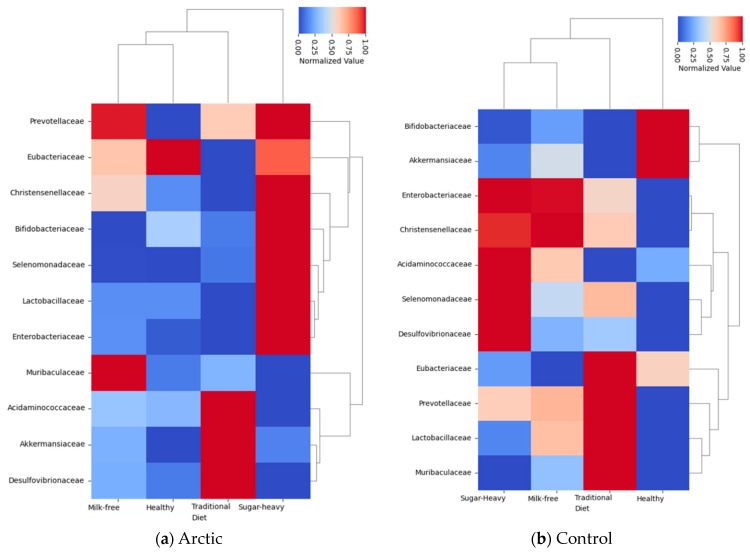
Heatmap of bacterial family levels according to diet type.

The next step was to find out how diets affect the number of representatives of various bacterial families. A complete list of the studied bacterial families is given in Supplementary Figures S1 and S2. In this study, it was decided to pay more attention to the bacterial families, the number of which significantly differed between the groups in the earlier analysis, without taking into account the diet (Table 8), and add several more families of *Lactobacillaceae*, *Bifidobacteriaceae,* and *Akkermansiaceae (*Figure 9*)*.

The final step was to conduct a statistical analysis between the two groups to compare the representatives of the bacterial families for the Arctic and control groups, taking into account food preferences. The test was performed using the Mann–Whitney criterion (see the Materials and Methods Section for details).

From the data collected, it can be noted that representatives of the bacterial families *Christensenellaceae* and *Muribaculaceae* dominated in the control group, both with a traditional diet and a milk-free diet. It can be assumed that, in this case, it is not only the diet that affects the number of representatives of these families, but also the environmental conditions of the region.

In the control group, representatives of the *Prevotellaceae*, *Enterobacteriaceae,* and *Comamonadaceae* families prevail when compared with the Arctic group (with a traditional diet). Representatives of the *Prevotellaceae* family are most often associated with proper nutrition; moreover, these bacteria have the most powerful anti-inflammatory effect [18].

It is also clear from the results obtained that the Arctic group has an increasing number of representatives of the families *Desulfovibrionaceae* (with a traditional diet) and *Enterobacteriaceae* (with a milk-free diet), which also corresponds to the results of early analysis (excluding diets, Table 8). Representatives of the genus *Desulfovibrio* are able to absorb sulfate and release hydrogen sulfide, which is known to cause a disruption of the intestinal barrier, which in turn leads to inflammatory gut diseases [19].

## 4. Discussion

The contingent living in the Arctic has a different diet from the residents of other regions of the Russian Federation. This is primarily due to the severe climatic conditions of the habitat and the meager variety of the diet.

It was decided to conduct two analyses, both of which focused on the Arctic groups (a combined group of indigenous and newcomer populations) and the control group. After observing no significant differences in the composition of gut microbiota between the native and newcomer populations, it was concluded to merge them into a single Arctic group. Given that the newcomer population had been living in the Arctic zone for at least a year, it can be suggested that their gut microbiota underwent significant changes, becoming similar to that of the native population. This result was highly anticipated, as the diet and living conditions of both the native and newcomer populations were comparable.

In the first analysis (Table 8), we compared the microbiota of the Arctic group and the control group without taking into account the diet of the participants, and, in the second analysis, we took into account the food preferences of the participants (Table 9). This analysis helped to determine the effect of the type of nutrition on the composition of the microbiota. The results showed that food preferences strongly influence the formation of microbiota composition in the Arctic and control groups.

For example, the results of this study show that the inhabitants of the Arctic, including those who arrived in these territories, significantly reduced the representation of the *Christensenellaceae* family relative to the control group, both without taking into account diet and when taking into account diet. During the research of this family, it was demonstrated that a decrease in the number of *Christensenellaceae* is one of the signs of microbiotic instability and is observed in people with obesity, type 2 diabetes mellitus, as well as those with an exacerbation of Crohn’s disease [20,21,22]. Other studies have also showed the role of this bacterial family in the prevention of intestinal inflammation and the enhancement of its barrier functions [23]. It is also worth noting that representatives of *Christensenellaceae* are involved in metabolic processes (for example, glucose metabolism), produce short-chain fatty acids, and are associated with a low BMI [24,25]. Bacteria of the *Christensenellaceae* family correlate with lower visceral fat deposits [26]. It has been demonstrated that the genus of bacteria *Christensenella* is rarely found in fat people, and, when administered to sterile mice, it prevents weight gain [24]. Considering that a decrease in the representation of the *Christensenellaceae* family is more often recorded in obese people, and the participants in our study do not suffer from this disease and have a BMI corresponding to normal body weight, it can be assumed that the direct cause of this is the meager diet of the Arctic group, without fresh vegetables and fruits, with a high content of highly processed food with a lot of complex carbohydrates and the presence of trans fats.

Concurrently with the decrease in the proportion of *Christensenellaceae*, the Arctic residents have an increase in the representation of *Selenomonadaceae* (Figure 8). Similar changes (decrease in *Christensenellaceae* with a simultaneous increase in *Selenomonadaceae*) were shown in [27] after partial replacement of cornstarch with whey permeate (lactose 80–85%) in the diet of cows, which suggests the relationship of these changes with the replacement of complex sugars in the diet with simple ones. However, it is worth noting that the group of microorganisms *Selenomonadaceae* is poorly studied, and different opinions have been put forward regarding their role in maintaining human health. For example, in [28], it was shown that an increase in the proportion of *Selenomonadaceae* is observed with an increased consumption of fermented foods. There is evidence of an increase in the representation of *Selenomonadaceae* in children with biliary atresia without concomitant cholangitis, which indirectly indicates the influence of nutrition on the number of this group of bacteria [29].

An increase in representatives of the *Acidaminococcaceae* family was found in the newcomer population. However, the accurate biological interpretation of this taxon is difficult due to its low level of research. In a study [30] on the connection between the composition of the human gut microbiota and a diet high in fatty acids, it was noted that the participants who consumed more total fatty acids and polyunsaturated fatty acids showed an increase in the number of representatives of the *Acidaminococcaceae* family in the large gut. The *Acidaminococcus* family ferments glutamate, which provides a substrate for oxidation in the intestinal epithelium and plays an important role in maintaining normal intestinal barrier function [31]. However, the researchers indicate the need for a larger experiment on other studied populations to continue to examine the association between *Acidaminococcus* and diet. It is worth noting that, in this analysis, taking into account the participants’ diets, there was no significant difference in the number of representatives of bacterial families.

There were differences in the bacterial composition of the gut microbiota of the newcomer population of the Arctic group and the control group. In particular, the newcomer population demonstrated an increase in the number of representatives of the *Desulfovibrionaceae* family relative to the control group. The work [32] demonstrated a positive association of the growth in the number of these microorganisms with the level of norepinephrine, one of the main stress hormones. A prevalence of the *Desulfovibrionaceae* family was found in individuals with impaired liver function, including non-alcoholic steatosis [33,34]. In experiments on mice, probiotic colonization of the gut with *Lactobacillus* and *Bifidobacterium* groups led to a decrease in the severity of steatosis, which suggests the participation of *Desulfovibrionaceae* in the pathogenesis of this disease [35]. In the study by Tagliabue et al. [36], an increase in the amount of *Desulfovibrio spp* in the intestinal microbiota was revealed in patients with glucose transporter deficiency syndrome 1 who adhere to keto diets from 1 to 3 months. Supposedly, *Desulfovibrio* spp. is involved in exacerbating the inflammatory condition of the intestinal mucosa associated with the consumption of animal fats. In parallel, the high prevalence of this group of microorganisms in people with normal and low BMI has been demonstrated, which makes it difficult to unambiguously interpret the effect of these bacteria on human health [37]. It is indicated in [38] that the families *Ruminococcaceae* and *Desulfovibrionaceae* are interrelated with the darkening of white adipose tissue when exposed to cold temperatures. It was observed that mice exposed to the cold had an increased content of representatives of these families [39]. The *Desulfovibrionaceae* family was previously associated with metabolic health [40]. There is a suggestion that the increase in *Desulfovibrionaceae* indicated in our work is associated with the adaptation of the alien population to Arctic conditions and reflects the high stress of these living conditions.

The number of representatives of the *Muribaculaceae* family prevails in the control group with traditional and milk-free diets. Earlier research has demonstrated that representatives of the *Muribaculaceae* family (otherwise known as the S24-7 family) develop ferments capable of breaking down complex carbohydrates, therefore, diets with a high fat content reduce the number of this family [41]. Also, a number of research studies have shown that the S24-7 family of fermenting bacteria has a positive effect on the course of type 1 diabetes and inflammatory arthritis [42,43]. It is probable that the indigenous and alien population of the Arctic zone consumes insufficient fresh vegetables and fruits, which contain fiber and dietary fiber, in comparison with the control group.

It is worth paying particular attention to the increase in representatives of the *Enterobacteriaceae* family among the native and newcomer population. These results were obtained in both analyses, both with and without the inclusion of diets. The largest number of representatives of the *Enterobacteriaceae* family was observed in the Arctic group following a milk-free diet. An increase in the number of representatives of the *Enterobacteriaceae* family is associated with obesity, inflammatory gut diseases, and metabolic disorders. In research [44], dysbiosis was recorded in mice with inflammatory gut disease in response to the inflammatory process, with a significant increase in the number of representatives of the *Enterobacteriaceae* family. Many representatives of the *Enterobacteriaceae* family enhance the inflammatory response of the human body. We can suggest that an increase in representatives of the *Enterobacteriaceae* family may be a sign of microbial imbalance and instability of the gut microbiome in residents of the Arctic region [45].

Another interesting family is *Prevotellaceae*. Representatives of this family prevail in the control group in comparison with the native population of the Arctic (analysis with and without taking into account diets). The *Prevotellaceae* family is associated with the gut microbiota of a healthy person and correlates with a diet rich in complex carbohydrates, plant-based foods, and fiber [46]. In our case, the largest number of representatives of the *Prevotellaceae* family was recorded in the control group following a traditional diet, which includes a high content of complex carbohydrates. The reduced number of representatives of *Prevotellaceae* in Arctic residents is probably due to the poor quality of the food itself and the lack of fresh vegetables and fruits. It is also worth noting the correlation of representatives of the *Prevotellaceae* and *Christensenellaceae* families.

The *Eubacteriaceae* family prevails in the control group in the correlation analysis (analysis with and without diets). There is sufficient evidence that an increase in fat or protein components with a simultaneous decrease in dietary fiber consumption, characteristic of a Western-type diet, negatively correlates with the number of *Eubacteriaceae* [47]. A reduction in the representation of this family relative to the control group was observed both among the indigenous inhabitants of the Arctic region and among the alien population compared with the control group of the middle latitudes of Russia. The presence of this bacterial family in the gut microbiome contributes to an adequate intake of complex dietary fibers, which is difficult to achieve in Arctic conditions. These changes in the microbial composition can negatively affect human health, since, with a reduced amount of *Eubacteriaceae*, fewer short-chain fatty acids are produced in the intestine, the positive effect of which has been shown in many studies [48].

It should be noted that the settlements of Tiksi and Chokurdakh are located in the Arctic zone of continuous permafrost, which directly affects the physical and psychological state of the population of these villages. Temperature stress caused by fluctuations in the ambient temperature affects physiological, biochemical, and molecular regulation. The exposure of mammals to temperatures below their thermoneutral threshold (+24 °C for humans) is considered a cold irritant [49]. Exposure to cold temperatures can affect the gut microbiota, which will affect human metabolic parameters. For example, in their study, [50] reported pronounced changes in the composition of the gut microbiota and metabolites derived from the gut microbiota, concomitant with increased permeability and inflammation of the gastrointestinal tract during 4 day military exercises conducted during the Arctic winter.

A number of studies have found that cold stress affects the state of the gut microbiota of animals. In the experiment reported in [51], the fecal microbiota of a cold-resistant breed of pigs (Mashen) and a cold-sensitive breed of pigs (Duroc-Landrace-Yorkshire) was transferred to microbe-free mice. After cold exposure (+4 °C), gut function and changes in the microbiota of mice were studied for 21 days. The results demonstrated that the transplantation of the microbiota of Mashen pigs stabilized the body temperature of mice whose adipose tissue weight and expression of thermogenin proteins, carnitine palmitoyltransferases (Cpt1b), and a gamma receptor coactivator, activated by the proliferator peroxisome, were significantly higher (*p* < 0.05) than those of the control group, which indicated an increase in the amount of brown adipose tissue. The results of the research on the intestinal structure and expression of serum inflammatory factors showed that mice undergoing fecal microbiota transplantation have an intact intestinal structure and a high expression of pro-inflammatory factors such as interleukin-4 (IL-4). The study of the fecal microbiome of mice characterized by 16S rRNA sequencing showed that microbiota transplantation led to dramatic changes in the composition of the microbiota, including an increase in the ratio of *Firmicutes* to *Bacteroidetes*. In addition, the distinctive features of *Firmicutes* in the microbiota between the two pig breeds have been identified. In mice with the implanted microbiota of Mashen pigs, representatives of the *Clostridiaceae* predominated, which suggests that these mice are characterized by a high degree of nutrient absorption. At the same time, the mice that received the microbiota of Duroc-Landrace-Yorkshire pigs had significantly more representatives of *Coriobacteriales* (*p* < 0.05). Finally, it was found that the content of propionic acid and butyric acid in rectal contents changed significantly in mice with the microbiota of cold-resistant pig breeds, and the amount of *Clostridium* and *Lachnospira* showed a significant correlation with changes in short-chain fatty acids. The results obtained demonstrate that pig fecal microbiota transplantation can mitigate changes in physiological and biochemical parameters in mice caused by exposure to cold temperatures by changing the composition of the gut microbiome and improving gut barrier function.

Recent evidence suggests that exposure to cold temperatures causes changes in the gut microbiota of mice, which may contribute to the physiological adaptation of the host to cold temperatures. In mammals, physiological adaptations after repeated or chronic exposure to cold temperatures include blunting the physiological response to cold, increased heat retention, and/or a more pronounced thermogenic reaction [52]. In animals, an increase in intestinal absorption capacity has also been reported after exposure to cold temperatures, and it is believed that this contributes to increased energy consumption to maintain an increased metabolic rate, which supports thermogenesis [53]. These adaptations can be partially facilitated by the gut microbiota. In particular, [2] reported that the gut microbiota of mice exposed to cold temperatures for 11–31 days showed no changes in community diversity. Nevertheless, a change in the relative abundance of several taxa of the gut microbiota of these mice was shown, some of which reflected features of the microbiota previously associated with obesity (for example, a change in the ratio of Firmicutes/Bacteroidetes and a decrease in the number of *Akkermansia*). Our study also shows a decrease in representatives of the families of bacteria in the Arctic group, namely *Christensenellaceae* and *Muribaculaceae*, which are associated with conditionally healthy people who lead a healthy lifestyle and adhere to a healthy diet.

There have been a number of studies conducted on the territory of the Russian Federation. T.N. Ivanova identified certain features of the gut microbiota of the inhabitants of the Far North in research. The author points out the fact that almost the entire population of Surgut has quantitative and qualitative deviations from the conventional norm in the composition of their microbiota. It is worth noting that such significant deviations were not observed among the residents of Perm [54]. Severe dysbiosis was detected in 17.5% of Surgut residents, characterized by a variety of types of dysbiosis and the release of a large amount of atypical and pathogenic microflora, which was 1.5 times higher than that observed in the Perm residents. At the same time, there was typically a decrease in the presence of *Lactobacillus* and the appearance of lactose-negative *E. coli* instead of a full-fledged enzymatic one. The residents of Surgut showed a 2.7-fold decrease in representatives of the genus *Enterococcus*, compared with the residents of Perm. In general, the residents of Surgut have 3.2 times more pathogens of intestinal infections, mainly due to *E. coli* and *Salmonella*.

In the works of Ya.A. Akhremenko et al., significant changes in the microbiota were found in the form of a reduced content of bifidobacteria and lactobacilli against the context of the activation of opportunistic and pathogenic microbiota in the colon of children from the city of Yakutsk [55]. However, in our study, there was a decrease in the number representatives of the *Lactobacillaceae* and *Bifidobacteriaceae* families in individuals from the Arctic group who adhere to a milk-free or traditional diet (Figure 9a).

If we generalize the obtained results, it can be noted that, among the residents of the Arctic zone, for both the native and the newcomer population, diversity is characterized by a decrease in representatives of families belonging to the so-called conditional norm of the intestinal microbiota, for example, the families *Christensenellaceae* and *Muribaculaceae*. These data are reproduced in the analysis both with and without taking into account diets. From this, it can be assumed that not only the type of nutrition, but also harsh climatic conditions, in the form of cold temperatures, long polar nights, and remoteness from large cities, can affect the physiological and psycho-emotional state of a person, which in turn can directly and indirectly affect the gut microbiota. At the same time, the Arctic group showed an increase in representatives of the bacterial families *Enterobacteriaceae* and *Desulfovibrionaceae*, which include a genus of opportunistic bacteria and bacteria associated with inflammatory processes in the human intestine. For example, the genera *Salmonella*, *Shigella*, and *Escherichia* belong to the *Enterobacteriaceae* family.

Thus, in the Arctic group, in comparison with the control group, in analyses taking into account and without taking into account food preferences, there was a significant decrease in representatives of bacterial families associated with a healthy lifestyle and an increase in representatives of bacterial families associated with inflammatory processes in the intestine. This imbalance can further lead to the instability of intestinal diversity and negatively affect human health [45].

Strengths:

Here, we provide a detailed analysis of gut microbiota specific to individuals living in the Arctic region characterized by extreme environmental conditions. This focus allows us to understand how cold temperatures and limited daylight could influence microbial composition and function, potentially affecting host health and adaptation mechanisms. Also, the inclusion of traditional dietary habits metadata in this research, which are often rich in animal fats, can provide insights into the physiological adaptations, such as enhanced lipid metabolism, and how these adaptations might influence overall health and disease susceptibility. During this study, we also tried to take into account psychological stressors, such as isolation and limited social interaction (which are common in Arctic regions), that can affect gut microbiota composition. It is particularly important for the further development of therapeutic strategies to mitigate stress-related dysbiosis through microbiota modulation.

Limitations:

Due to the sparse population in the Arctic region, studies like this often involve small cohorts, which can limit the statistical power and generalizability of the findings. This constraint makes it challenging to draw broad conclusions about the gut microbiota of Arctic populations overall and suggests a narrow interpretation of the results. Another limitation that could be taken into account is the initial assumption of average ethnic backgrounds. The absence of a specific evaluation can overlook the genetic and local metabolic diversity in the gut, which may influence microbiota composition. Genetic and ethnicity-specific factors can affect microbial communities and add variability to the data. Also, the 16S rRNA approach provides a wide snapshot of the microbial community at a given time, which may not capture the dynamic changes observed in microbiota composition over different seasons or the life cycle of patients.

## 5. Conclusions

In the course of this work, bacterial families characteristic of people living in the Arctic zone of the Far Eastern region of the Russian Federation were identified. It is worth noting that the number of such studies is extremely small, therefore, it is of great value. A large number of people live in the permafrost zone, both permanently and provisionally. Poor diet, difficult climatic conditions, and problems with logistics and medical care can have a strong impact on the health of this population.

In the course of our study, a unique collection of samples was collected from conditionally healthy men of different nationalities who were born or moved to live in fairly remote villages in the Far Eastern region of the Russian Federation. It was noted that, in the newcomers population, during a year of stay in the Arctic zone, the gut microbiota became almost completely similar to the gut microbiota of the native population, which confirms the view of the microbiota as a dynamic system capable of adapting to changing environmental conditions and to the type of nutrition in a fairly short time. We also saw that the microbial composition of Arctic residents differs from the gut microbiota of residents of temperate latitudes (Omsk).

In our study, an extensive questionnaire was conducted among the participants, where many questions were included about the dietary preferences of the study participants, which allowed us to further divide them into groups according to their diets. From this analysis, it can be seen that most of the participants adhere to the so-called traditional diet for this region. They consume a large number of low-cost products, obtain animal protein from poultry and canned food, and also eat a small number of fresh vegetables and fruits. Such a diet is due to the social status of the study participants and the climatic and geographical features of the region (difficulties in agriculture). With such a diet, we observe a decrease in representatives of the *Christensenellaceae*, *Muribaculaceae*, *Eubacteriaceae*, and *Prevotellaceae* families and an increase in representatives of the *Enterobacteriaceae* and *Desulfovibrionaceae* families among Arctic residents. This imbalance may cause this population to develop various diseases in the future, including chronic diseases such as obesity, intestinal dysbiosis, inflammatory bowel diseases, and type 2 diabetes.

In the future, we would like to continue studying the bacterial composition of the intestine in people living in the Arctic zone, expand the sample of participants to include women and children, and obtain biomaterials from people with various diseases. Such research can help us to adjust a special diet for Arctic residents (both newcomers and natives), as well as develop a special probiotic that can help the Arctic population to better adapt to living in difficult climatic conditions.

## Figures and Tables

**Table 1:** Composition of the studied groups.

	Control n = 51	Native n = 98	Newcomer n = 59
	Age		
average value	51.02	48.09	44.98
	BMI		
average value	25.65	26.63	25.51

**Table 2:** The results of multidimensional testing for differences between groups.

	Group	*p*-Value
RDP	Control//newcomers	0
	Control//native	1.110223 × 10^−16^
	Newcomers//native	0.0011
	Arctic//control	0
SILVA	Control//newcomers	0
	Control//native	0
	Newcomers//native	6.063054 × 10^−7^
	Arctic//control	0

**Table 3:** Results of permutation analysis of variance (PERMANOVA).

	RDP	SILVA
Weighted UniFrac	0.008	0.011
Unweighted UniFrac	0.059	0.036

**Table 4:** Mann–Whitney and LEfSe test results for RDP data.

RDP			
Control and Native			
**Family**	**M-У (α = 0.05)**	**LEfSe (α = 0.05)**	**Enrichment**
*Eubacteriaceae*	0.0002	0.0001	control
*Enterobacteriaceae*	0.0025	0.0318	native
*Odoribacteraceae*	0.0171	0.004	control
*Erysipelotrichaceae*	0.027	>0.1	native
*Methanobacteriaceae*	0.0362	>0.1	native
*Christensenellaceae*	0.038	0.0012	control
*Prevotellaceae*	>0.05	0.03	control
*Barnesiellaceae*	>0.05	0.03	control
*Muribaculaceae*	>0.05	0.0003	control
Control and Newcomer			
**Family**	**M-У (α = 0.05)**	**LEfSe (α = 0.05)**	**Enrichment**
*Eubacteriaceae*	0.0001	8.850962 × 10^−5^	control
*Christensenellaceae*	0.0016	1.359842 × 10^−4^	control
*Selenomonadaceae*	0.0045	2.018457 × 10^−2^	newcomer
*Desulfovibrionaceae*	0.00502	3.851101 × 10^−2^	newcomer
*Enterobacteriaceae*	0.0181	>0.1	newcomer
*Ruminococcaceae*	0.022	2.221230 × 10^−2^	control
*Lactobacillaceae*	0.031	>0.1	newcomer
*Muribaculaceae*	>0.05	1.958630 × 10^−2^	newcomer
Newcomer and Native			
**Family**	**M-У (α = 0.05)**	**LEfSe (α = 0.05)**	**Enrichment**
*Methanobacteriaceae*	0.024	>0.1	native
*Ruminococcaceae*	0.04	>0.1	native
*Erysipelotrichaceae*	0.049	>0.1	native
*Acidaminococcaceae*	>0.05	0.04892970	newcomer
*Actinomycetaceae*	>0.05	0.02466564	newcomer
Control and Arctic			
**Family**	**M-У (α = 0.05)**	**LEfSe (α = 0.05)**	**Enrichment**
*Eubacteriaceae*	2.846091 × 10^−5^	1.495706 × 10^−5^	control
*Enterobacteriaceae*	2.073474 × 10^−3^	2.480785 × 10^−2^	Arctic
*Selenomonadaceae*	3.307885 × 10^−3^	4.984589 × 10^−2^	Arctic
*Christensenellaceae*	5.094336 × 10^−3^	5.995476 × 10^−5^	control
*Lactobacillaceae*	1.138849 × 10^−2^	>0.1	Arctic
*Desulfovibrionaceae*	1.449099 × 10^−2^	>0.1	Arctic
*Synergistaceae*	1.571793 × 10^−2^	>0.1	Arctic
*Prevotellaceae*	4.633777 × 10^−2^	2.172070 × 10^−2^	control
*Muribaculaceae*	>0.05	2.589878 × 10^−4^	control
*Odoribacteraceae*	>0.05	9.994337 × 10^−3^	control

**Table 5:** Mann–Whitney and LEfSe test results for SILVA data.

SILVA			
Control and Native			
**Family**	**M-У (α = 0.05)**	**LEfSe (α = 0.05)**	**Enrichment**
*Christensenellaceae*	0.001	0.0007	control
*Enterobacteriaceae*	0.001	0.0192	native
*Marinifilaceae*	0.018	0.006	control
*Synergistaceae*	0.028	>0.1	native
*Selenomonadaceae*	0.029	>0.1	native
*Erysipelotrichaceae*	0.036	>0.1	native
*Succinivibrionaceae*	0.037	>0.1	native
*Prevotellaceae*	>0.05	0.041	control
*Muribaculaceae*	>0.05	0.009	control
*Barnesiellaceae*	>0.05	0.037	control
*Clostridia_vadinBB60_group*	>0.05	0.0007	control
Control and Newcomer			
**Family**	**M-У (α = 0.05)**	**LEfSe (α = 0.05)**	**Enrichment**
*Christensenellaceae*	1.530570 × 10^−6^	8.777662 × 10^−7^	control
*[Eubacterium]_coprostanoligenes_group*	2.618761 × 10^−4^	2.463391 × 10^−4^	control
*Selenomonadaceae*	2.948728 × 10^−3^	1.978220 × 10^−2^	newcomer
*Desulfovibrionaceae*	3.443668 × 10^−3^	3.647862 × 10^−2^	newcomer
*Enterobacteriaceae*	9.123330 × 10^−3^	4.215100 × 10^−2^	newcomer
*Synergistaceae*	0.03	>0.1	newcomer
*Succinivibrionaceae*	0.03	>0.1	newcomer
*Ruminococcaceae*	>0.05	3.725840 × 10^−2^	control
*Muribaculaceae*	>0.05	8.371989 × 10^−3^	control
*Clostridia_vadinBB60_group*	>0.05	9.287814 × 10^−4^	control
*Anaerovoracaceae*	>0.5	3.772745 × 10^−2^	control
Newcomer and Native			
**Family**	**M-У (α = 0.05)**	**LEfSe (α = 0.05)**	**Enrichment**
*[Eubacterium]_coprostanoligenes_group*	0.006	0.006	native
*Christensenellaceae*	0.006	0.006	native
*Acidaminococcaceae*	0.047	0.04	newcomer
Control and Arctic			
**Family**	**M-У (α = 0.05)**	**LEfSe (α = 0.05)**	**Enrichment**
*Christensenellaceae*	1.272051 × 10^−5^	8.384912 × 10^−6^	control
*Enterobacteriaceae*	1.161358 × 10^−3^	1.363322 × 10^−2^	Arctic
*Selenomonadaceae*	5.398135 × 10^−3^	4.712879 × 10^−2^	Arctic
*[Eubacterium]_coprostanoligenes_group*	1.858210 × 10^−2^	1.576306 × 10^−2^	Arctic
*Desulfovibrionaceae*	2.315924 × 10^−2^	>0.1	Arctic
*Erysipelotrichaceae*	4.488905 × 10^−2^	>0.1	Arctic
*Prevotellaceae*	>0.05	3.618655 × 10^−2^	control
*Muribaculaceae*	>0.05	2.967150 × 10^−3^	control
*Marinifilaceae*	>0.05	1.790566 × 10^−2^	control

**Table 6:** Accuracy of the RF model for all analysis groups.

Accuracy	Native and Control	Newcomer and Control	Arctic and Control	Native and Newcomer
RDP	90%	88%	94.5%	56%
SILVA	91%	85%	93%	58%

**Table 7:** Results of correlation analysis.

**RDP**		
**Family 1 + Family 2**	**Arctic**	**Control**
Changes in the type of interaction		
*Eggerthellaceae + Acidaminococcaceae*	−0.21	0.32
*Sutterellaceae + Prevotellaceae*	−0.16	0.31
*Akkermansiaceae + Rikenellaceae*	−0.16	0.35
*Acidaminococcaceae + Lachnospiraceae*	−0.30	0.28
Weakening of the connection among Arctic residents		
*Bifidobacteriaceae + Pasteurellaceae*	0.30	0.61
*Methanobacteriaceae + Eubacteriaceae*	0.20	0.53
*Clostridiales Incertae Sedis XIII + Christensenellaceae*	0.19	0.41
*Methanobacteriaceae + Bacteroidaceae*	−0.19	−0.33
*Clostridiales_Incertae Sedis XIII + Barnesiellaceae*	−0.20	−0.38
Increased interconnection among Arctic residents		
*Desulfovibrionaceae + Acidaminococcaceae*	0.67	0.55
*Odoribacteraceae + Bacteroidaceae*	0.49	0.33
**SILVA**		
Changes in the type of interaction		
*Acidaminococcaceae + Lachnospiraceae*	−0.30	0.28
*Saccharimonadaceae + Ruminococcaceae*	−0.16	0.34
Weakening of the connection among Arctic residents		
*Bifidobacteriaceae + Saccharimonadaceae*	0.26	0.50
*Bifidobacteriaceae + Pasteurellaceae*	0.30	0.61
*Christensenellaceae + Oscillospiraceae*	0.22	0.43
*Clostridia UCG 014 + Clostridiaceae*	0.30	0.54
*Clostridia_vadinBB60_group + Izemoplasmatales*	0.33	0.53
*Eggerthellaceae + Erysipelatoclostridiaceae*	0.29	0.44
*Methanobacteriaceae + Bacteroidaceae*	−0.19	−0.33
*Methanobacteriaceae + Eubacterium coprostanoligenes group*	0.27	0.55
*Oscillospiraceae + Muribaculaceae*	0.17	0.47
*Rikenellaceae + Lachnospiraceae*	−0.21	−0.40
*Ruminococcaceae + Prevotellaceae*	−0.22	−0.46
Increased interconnection among Arctic residents		
*Clostridia UCG 014 + Methanobacteriaceae*	0.58	0.28
*Marinifilaceae + Bacteroidaceae*	0.49	0.33

**Table 8:** Results of three methods of use (Mann–Whitney testing, LEfSe, and Random Forest).

**RDP**					
	**Mann–Whitney U test**	**LEfSe**	**Random Forest**	**n**	**Abundance**
control and native	Eubacteriaceae	Eubacteriaceae	Eubacteriaceae	3	control
	Enterobacteriaceae	Enterobacteriaceae	Enterobacteriaceae	3	native
	Odoribacteraceae	Odoribacteraceae		2	control
	Christensenellaceae	Christensenellaceae	Christensenellaceae	3	control
		Prevotellaceae	Prevotellaceae	2	control
		Muribaculaceae	Muribaculaceae	2	control
control and newcomer	Eubacteriaceae	Eubacteriaceae	Eubacteriaceae	3	control
	Christensenellaceae	Christensenellaceae	Christensenellaceae	3	control
	Selenomonadaceae	Selenomonadaceae		2	newcomer
	Desulfovibrionaceae	Desulfovibrionaceae		2	newcomer
	Enterobacteriaceae		Enterobacteriaceae	2	newcomer
	Ruminococcaceae	Ruminococcaceae	Ruminococcaceae	3	control
native and newcomer	Ruminococcaceae		Ruminococcaceae	2	native
		Acidaminococcaceae	Acidaminococcaceae	2	newcomer
control and arctic	Eubacteriaceae	Eubacteriaceae	Eubacteriaceae	3	control
	Enterobacteriaceae	Enterobacteriaceae	Enterobacteriaceae	3	arctic
	Selenomonadaceae	Selenomonadaceae		2	arctic
	Prevotellaceae	Prevotellaceae	Prevotellaceae	3	control
	Christensenellaceae	Christensenellaceae	Christensenellaceae	3	control
		Muribaculaceae	Muribaculaceae	2	Control
**S** **ILVA**					
	**Mann–Whitney U test**	**LEfSe**	**Random Forest**	**n**	**Abundance**
control and native	Christensenellaceae	Christensenellaceae	Christensenellaceae	3	control
	Enterobacteriaceae	Enterobacteriaceae		2	native
	Marinifilaceae	Marinifilaceae		2	control
		Prevotellaceae	Prevotellaceae	2	control
		Muribaculaceae	Muribaculaceae	2	control
		Barnesiellaceae	Barnesiellaceae	2	control
control and newcomer	Christensenellaceae	Christensenellaceae	Christensenellaceae	3	control
	[Eubacterium]_coprostanoligenes_group	[Eubacterium]_coprostanoligenes_group	[Eubacterium]_coprostanoligenes_group	3	control
	Selenomonadaceae	Selenomonadaceae		2	newcomer
	Desulfovibrionaceae	Desulfovibrionaceae		2	newcomer
	Enterobacteriaceae	Enterobacteriaceae		2	newcomer
		Muribaculaceae	Muribaculaceae	2	control
native and newcomer	[Eubacterium]_coprostanoligenes_group	[Eubacterium]_coprostanoligenes_group	[Eubacterium]_coprostanoligenes_group	3	native
	Christensenellaceae	Christensenellaceae		2	native
	Acidaminococcaceae	Acidaminococcaceae	Acidaminococcaceae	3	newcomer
control and arctic	Christensenellaceae	Christensenellaceae	Christensenellaceae	3	control
	Enterobacteriaceae	Enterobacteriaceae		2	arctic
		Prevotellaceae	Prevotellaceae	2	control
		Muribaculaceae	Muribaculaceae	2	control
	Selenomonadaceae	Selenomonadaceae		2	arctic
	[Eubacterium]_coprostanoligenes_group	[Eubacterium]_coprostanoligenes_group		2	control

**Table 9:** The result of statistical analysis for each diet between the Arctic and control groups.

Diet	Family	*p*-Value	Abundance
Milk-free	*Christensenellaceae*	0.041884	control
Traditional	*Christensenellaceae*	0.003175	control
Milk-free	*Enterobacteriaceae*	0.030336	Arctic
Traditional	*Desulfovibrionaceae*	0.033056	Arctic
Traditional	*Comamonadaceae*	0.01766	control
Traditional	*Eubacteriaceae*	0.000047	control
Traditional	*Prevotellaceae*	0.003326	control
Milk-free	*Muribaculaceae*	0.042727	control
Traditional	*Muribaculaceae*	0.00033	control

## Data Availability

The datasets used and analyzed in the present study are available from the corresponding author on reasonable request.

## Supplementary Materials

The following supporting information can be downloaded at: https://www.mdpi.com/article/10.3390/biomedicines12112472/s1, Figure S1: Heatmap of bacterial family levels according to diet type for the control group; Figure S2: Heatmap of bacterial family levels according to diet type for the Arctic group; Table S1: The list of families identified during the analysis; Microbiota Donor Questionnaire.
