# Supplementary material for: Characteristics of the Gut Microbiota Composition of the Arctic Zone Residents in the Far Eastern Region
Source: Biomedicines. 2024 Oct 28;12(11):2472. doi: 10.3390/biomedicines12112472 (PMC11591809; doi:10.3390/biomedicines12112472)
Supplement: Supplementary file 1 [file biomedicines-12-02472-s001.zip › biomedicines-3197276-supplementary.pdf]

Supplementary materials.

Table S1. The list of families identified during the analysis.

	**natives**	newcomers	control
Acholeplasmataceae	1,00	0,33	1,00
Acidaminococcaceae	0,87	1,00	0,60
Actinomycetaceae	0,00	1,00	0,50
Aerococcaceae	0,68	1,00	0,13
Aeromonadaceae	0,00	1,00	0,00
Akkermansiaceae	0,94	0,80	1,00
Anaeroplasmataceae	1,00	0,00	1,00
Arcobacteraceae	0,00	0,00	1,00
Atopobiaceae	0,44	1,00	0,06
Bacillaceae	0,00	0,00	0,00
Bacillales_Incertae_Sedis_XI	0,00	0,00	0,00
Bacteroidaceae	0,89	1,00	0,81
Barnesiellaceae	0,82	0,91	1,00
Bdellovibrionaceae	0,00	0,00	1,00
Bifidobacteriaceae	0,81	1,00	0,43
Burkholderiaceae	0,00	1,00	0,00
Campylobacteraceae	0,00	0,00	1,00
Cardiobacteriaceae	0,00	0,00	0,00
Carnobacteriaceae	1,00	1,00	0,50
Catabacteriaceae	0,37	0,00	1,00
Caulobacteraceae	0,00	0,00	0,00
Chitinophagaceae	0,00	1,00	0,00
Chloroplast	0,11	0,04	1,00
Christensenellaceae	0,30	0,39	1,00
Chromatiaceae	0,00	1,00	0,00
Clostridiaceae	0,88	0,32	1,00
Clostridiales_Incertae_Sedis_XI	0,00	0,00	1,00
Clostridiales_Incertae_Sedis_XIII	0,72	0,51	1,00
Comamonadaceae	0,22	0,33	1,00
Coriobacteriaceae	1,00	0,83	0,89
Corynebacteriaceae	0,00	0,00	0,00
Cytophagaceae	0,00	1,00	0,00
Desulfobacteraceae	0,42	1,00	0,31
Desulfovibrionaceae	0,80	1,00	0,47
Eggerthellaceae	0,74	0,56	1,00
Elusimicrobiaceae	0,22	0,29	1,00
Enterobacteriaceae	1,00	0,84	0,85
Enterococcaceae	1,00	0,17	0,17
Erysipelatoclostridiaceae	0,06	0,09	1,00
Erysipelotrichaceae	1,00	0,75	0,63
Eubacteriaceae	0,62	0,60	1,00
Family_I	1,00	0,00	0,00
Flavobacteriaceae	0,00	1,00	0,00

Fusobacteriaceae	1,00	0,25	0,00
Gracilibacteraceae	0,64	0,72	1,00
Hafniaceae	0,50	1,00	0,00
Kiloniellaceae	0,07	0,06	1,00
Lachnospiraceae	1,00	0,98	0,93
Lactobacillaceae	1,00	0,97	0,37
Leptotrichiaceae	0,00	0,00	0,00
Marinilabiliaceae	1,00	0,00	0,00
Metamycoplasmataceae	1,00	0,00	0,00
Methanobacteriaceae	1,00	0,50	0,76
Methanomassiliicoccaceae	0,00	0,00	1,00
Methylobacteriaceae	0,73	1,00	0,00
Micrococcaceae	0,00	0,00	0,00
Moraxellaceae	0,00	0,00	0,00
Morganellaceae	0,00	0,00	0,00
Muribaculaceae	0,09	0,28	1,00
Mycoplasmataceae	1,00	0,00	0,00
Neisseriaceae	0,00	0,00	0,00
Odoribacteraceae	0,71	0,93	1,00
Oligosphaeraceae	0,00	0,00	1,00
Oxalobacteraceae	1,00	1,00	1,00
Paenibacillaceae	0,00	0,00	0,00
Pasteurellaceae	0,38	1,00	0,32
Peptococcaceae	1,00	0,75	0,50
Peptoniphilaceae	0,00	0,00	0,00
Peptostreptococcaceae	0,56	0,92	1,00
Poaceae	0,09	0,01	1,00
Porphyromonadaceae	0,82	1,00	0,59
Prevotellaceae	0,79	0,89	1,00
Propionibacteriaceae	0,00	0,00	0,00
Proteinivoraceae	0,00	1,00	0,00
Pseudomonadaceae	1,00	0,00	0,00
Puniceicoccaceae	0,00	0,00	1,00
Rhodobacteraceae	0,00	0,00	0,00
Rikenellaceae	0,76	1,00	0,77
Ruminococcaceae	1,00	0,86	0,96
Selenomonadaceae	0,94	1,00	0,44
Sphaerochaetaceae	0,00	0,00	1,00
Sphingobacteriaceae	1,00	0,77	0,94
Sphingomonadaceae	0,00	0,00	0,00
Spirochaetaceae	0,00	0,00	1,00
Spiroplasmataceae	0,37	1,00	0,56
Staphylococcaceae	0,00	0,00	0,00
Streptococcaceae	1,00	0,91	0,69
Streptomycetaceae	1,00	0,55	0,94
Succinivibrionaceae	0,90	1,00	0,45
Sutterellaceae	1,00	0,94	0,53

Synergistaceae	1,00	0,29	0,11
Syntrophomonadaceae	0,00	0,00	1,00
Thermaceae	0,00	0,00	1,00
Vallitaleaceae	0,00	0,00	0,00
Veillonellaceae	0,90	1,00	0,64
Victivallaceae	0,45	0,25	1,00
Weeksellaceae	0,00	1,00	0,00
Xanthomonadaceae	0,00	1,00	0,00
Yersiniaceae	0,15	1,00	0,00

The evaluation of abundance fluctuations for each detected bacterial family which provides a means to compare the abundances among the analyzed patient cohorts simultaneously. In this section, we present the distribution of detected bacterial families across the analyzed cohorts. Each column represents the relative abundance of each bacterial family for the three patient groups: natives, newcomers, and control. The calculated values range from 0 to 1, where values close to 0 indicate minimal abundance and values close to 1 indicate maximum abundance for each bacterial family.

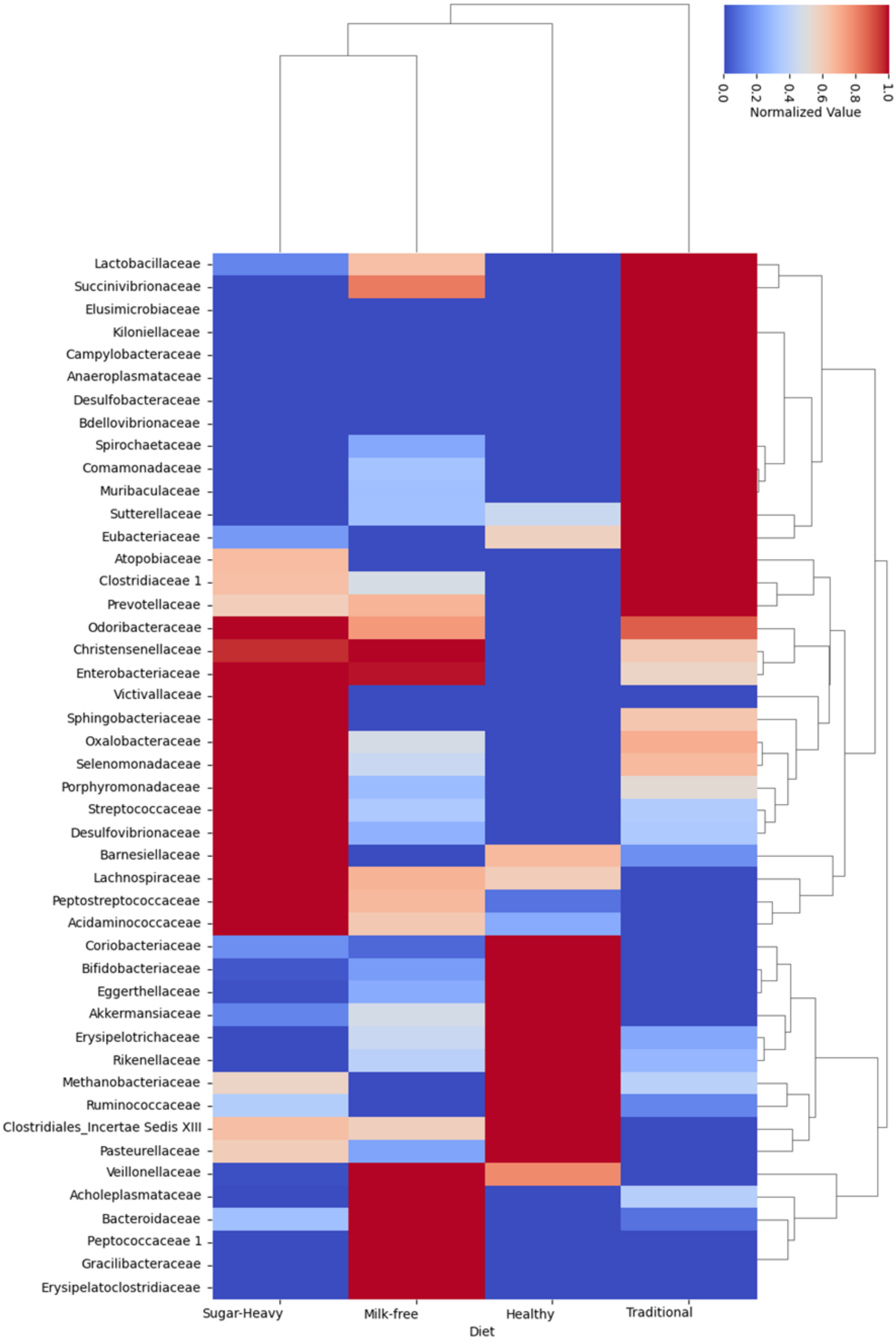

Figure S1. Heatmap of bacterial family levels according to diet type for the control group

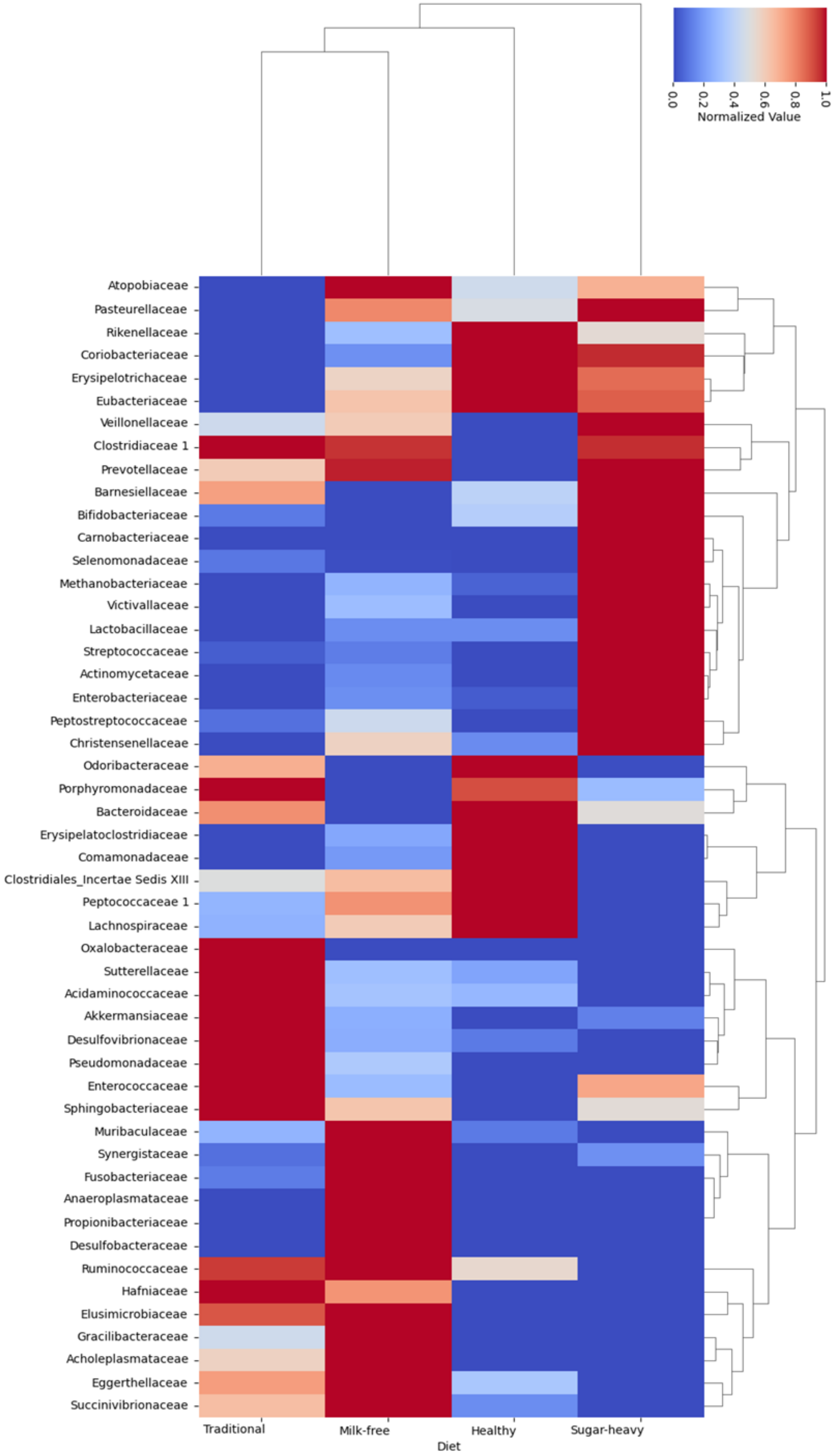

Figure S2. Heatmap of bacterial family levels according to diet type for the Arctic group
