# Supplementary material for: Characteristics of the Gut Microbiota Composition of the Arctic Zone Residents in the Far Eastern Region
Source: Biomedicines. 2024 Oct 28;12(11):2472. doi: 10.3390/biomedicines12112472 (PMC11591809; doi:10.3390/biomedicines12112472)
Supplement: Supplementary file 1 [file biomedicines-12-02472-s001.zip › Microbiota Donor Questionnaire.pdf]

Microbiota Donor Questionnaire

Please fill out the information in this questionnaire based on the average values of the consumption of certain foods over the past month. Please choose only one answer option when answering the question (except for questions where the opposite is indicated).

Place for the label

1. 1. Information about the donor

1.5. Gender ______________________________

1.6. Age ______________________________

1.7. Human height ______________________________

1.8. Date of birth ______________________________

1.9. Place of birth (country, city) ______________________________

1.10. Weight ______________________________

1.11. Phone number ______________________________

1.12. Body temperature (°C) ______________________________

1.13. Blood pressure (upper) ______________________________

1.14. Blood pressure (lower) ______________________________

1.15. Heart rate, beats per minute ______________________________

1.16. Condition of the skin

○ healthy ○ other (write) ______________________________

1.17. Condition of the Oropharynx:

○ healthy ○ other (write) ______________________________

2. Food preferences

2.1. Do you prefer any special diet?
- ○ yes (write) ______________________________
- ○ no

2.2. What kind of diet do you follow?
- ○ vegetarianism
- ○ veganism
- ○ raw food diet
- ○ traditional diet
- ○ a different type of food (write)______________________________

2.3. How often do you eat during the day?
- ○ less than once a day
- ○ 1-2 times a day
- ○ 3 times a day
- ○ 4 times and more often

2.4. 2.4. How often do you eat fresh fruits and/or berries?

- ○ less than once a week
- ○ 1-2 times a week
- ○ 3-5 times a week
- ○ more than 5 times a week

2.5. How often do you eat green vegetables?

- ○ less than once a week
- ○ 1-2 times a week
- ○ 3-5 times a week
- ○ more than 5 times a week

2.6. How often do you eat potatoes, white rice, pasta?

- ○ less than once a week
- ○ 1-2 times a week
- ○ 3-5 times a week
- ○ more than 5 times a week

2.7. How often do you eat meat?

- ○ less than once a week
- ○ 1-2 times a week
- ○ 3-5 times a week
- ○ more than 5 times a week

2.8. What kind of meat do you prefer?

- ○ birds (chicken, duck)
- ○ beef (cow)
- ○ pork
- ○ lamb
- ○ other _____________________

2.9. How often do you eat semi-finished products: sausages, salami and canned food?

- ○ less than once a week
- ○ 1-2 times a week
- ○ 3-5 times a week
- ○ more than 5 times a week

2.10. Do you eat foods high in fiber: cereals and legume?

- ○ less than once a week
- ○ 1-2 times a week
- ○ 3-5 times a week
- ○ more than 5 times a week

2.11. Do you eat foods or dietary supplements rich in vitamin D: fish (especially salmon), cod liver or eggs?

- ○ less than once a week
- ○ 1-2 times a week
- ○ 3-5 times a week
- ○ more than 5 times a week

2.12. Do you eat foods or dietary supplements rich in vitamin E: vegetable oils or nuts?

- ○ less than once a week
- ○ 1-2 times a week

- 3-5 times a week
- more than 5 times a week

2.13. Do you eat foods or dietary supplements rich in vitamin C: sea buckthorn, paprika, currant or citrus fruits?
- less than once a week
- 1-2 times a week
- 3-5 times a week
- more than 5 times a week

2.14. Do you eat foods or dietary supplements rich in omega-3 polyunsaturated fatty acids: walnuts, salmon, olive or linseed oil?
- less than once a week
- 1-2 times a week
- 3-5 times a week
- more than 5 times a week

2.15. How often do you eat sugar, sweet pastries and sweet dishes?
- less than once a week
- 1-2 times a week
- 3-5 times a week
- more than 5 times a week

2.16. How often do you drink coffee?
- 1-2 times a week
- 3-5 times a week
- every day
- more than once a day
- less than once a week

2.17. How often do you additionally salt your food?
- almost always
- sometimes
- almost never

2.18. Do you regularly eat foods rich in saturated fatty acids: butter, palm oil, lard, cream, cheese, meat?
- less than once a week
- 1-2 times a week
- 3-5 times a week
- more than 5 times a week

2.19. How often do you eat boiled or fried fish?
- less than once a week
- 1-2 times a week
- 3-5 times a week
- more than 5 times a week

2.20. Do you regularly eat foods containing trans-unsaturated fats: margarine, pastries, cookies and crackers, fried food?
- less than once a week
- 1-2 times a week
- 3-5 times a week
- more than 5 times a week

2.21. Do you regularly eat foods or dietary supplements rich in iron: beans, hazelnuts, mushrooms, beef liver, apples?
- ◯ less than once a week
- ◯ 1-2 times a week
- ◯ 3-5 times a week
- ◯ more than 5 times a week

2.22. Do you regularly eat foods or dietary supplements rich in iron: beans, hazelnuts, mushrooms, beef liver, apples?
- ◯ less than once a week
- ◯ 1-2 times a week
- ◯ 3-5 times a week
- ◯ more than 5 times a week

2.23. Do you regularly eat foods or dietary supplements rich in zinc: yeast, sesame seeds, pumpkin seeds, peanuts?
- ◯ less than once a week
- ◯ 1-2 times a week
- ◯ 3-5 times a week
- ◯ more than 5 times a week

2.24. Do you eat a lot of foods rich in fructose: honey, sweet dishes, sweeteners?
- ◯ less than once a week
- ◯ 1-2 times a week
- ◯ 3-5 times a week
- ◯ more than 5 times a week

2.25. How often do you eat fast food?
- ◯ less than once a week
- ◯ 1-2 times a week
- ◯ 3-5 times a week
- ◯ more than 5 times a week

2.26. Have you followed any religious fasts, diets and other dietary restrictions in the last 1 month?
- ◯ yes
- ◯ no

2.27. How often do you eat fermented milk products (kefir, yogurt and etc.)?
- ◯ never
- ◯ less than once a week
- ◯ 1-2 times a week
- ◯ 3-5 times a week
- ◯ more than 5 times a week

2.28. How often do you consume milk?
- ◯ never
- ◯ less than once a week
- ◯ 1-2 times a week
- ◯ 3-5 times a week
- ◯ more than 5 times a week

2.29. Do you have any food allergies or food intolerance?
- ◯ yes (write) ____

○ no

2.30. Do you regularly consume any kind of sports nutrition?
○ no ○ yes, specifically:
☐ proteins
☐ gainers
☐ BCAA
☐ fat burners
☐ other (write) ____________________

2.31. Do you use medications to lose body weight?
○ yes
○ no

2.32. Do you keep a food diary on a regular basis? Can you provide downloads or copies for the last month (for example, from FatSecret, MyFitnessPal applications)?
○ yes
○ no

2.33. Do you want to explain anything about the diet you follow?
____
____
____
____
____

2.34. Have you been ill with infectious diseases?
○ no ○ yes, specifically:

☐ HIV	☐ syphilis	☐ toxoplasmosis
☐ hepatitis B	☐ gonorrhea	☐ salmonellosis
☐ hepatitis C	☐ tuberculosis	☐ other

____
____
____

2.35. Have you been ill with infectious diseases in the last 6 months::
○ no ○ yes, specifically:

☐ candidiasis	☐ parasitosis
☐ urinary tract infections	☐ acute intestinal infection

☐ other: ____________________
____
____

2.36. Have you had any sexually transmitted diseases in the last 2 months?
○ no ○ yes, specifically:

☐ chlamydia	☐ trichomoniasis
☐ genital herpes	☐ other: ____________

____
____
____
____

2.37. Have you been vaccinated in the last six months?

○ no ○ yes, specifically:

- ☐ hepatitis B ☐ influenza ☐ tularemia ☐ rubella
- ☐ tetanus ☐ brucellosis ☐ tuberculosis ☐ poliomyelitis
- ☐ diphtheria ☐ plague ☐ VZV ☐ rabies
- ☐ pertussis ☐ other vaccines: ___________________________________

____

____

2.38. Have you been injected with one of the listed medications?

○ no ○ yes, specifically:

- ☐ anti-tetanus serum
- ☐ immunoglobulin against hepatitis B

2.39. Have you had any operations on the gastrointestinal tract?

○ no ○ yes, specifically:

- ☐ appendectomy ☐ cholecystectomy
- ☐ removal of a section of the colon outside through an opening in the stomach (colostomy)
- ☐ резекция или протезирование любого из отделов желудочно-кишечного тракта

2.40. Have there been any surgical interventions unrelated to the gastrointestinal tract in the last 6 months?

○ yes (write) ____

○ no

2.41. Have you had random sexual connections in the last 6 months?

○ yes

○ no

2.42. Have you been a blood donor or had an organ and tissue transplant in the last 6 months?

○ yes

○ no

2.43. Have you had a blood transfusion during the year?

○ yes

○ no

2.44. Have there been intravenous injections or taking blood from a vein in the last 6 months?

○ yes

○ no

2.45. Have you had tattoos, piercings and acupuncture done in the last 6 months?

○ yes

○ no

2.46. Have you traveled to tropical countries in the last 6 months?

○ yes

○ no

2.47. Do you work in the medical area?

○ yes

○ no

2.48. Have you been in detention for more than 72 hours?

○ yes

○ no

2.49. 2.47. Do you breed pets, train animals professionally, or are you an employee/an active volunteer at an animal nursery or shelter? If at least one point is correct, check "yes".

○ yes
○ no

2.50. Do you have any of the listed types of diseases?
○ no ○ yes, specifically:
☐ congenital abnormalities of the gastrointestinal tract (write) ____________________
☐ chronic diseases of the gastrointestinal tract (write) ____________________
☐ hiatus hernia
☐ celiac disease (gluten intolerance)
☐ chronic systemic autoimmune diseases (write) ____________________
☐ colon cancer
☐ other tumors of the gastrointestinal tract (write) ____________________

2.51. Have there been recent manifestations of the following symptoms (no more than 14 days ago)?
○ no ○ yes, specifically :
☐ diarrhea ☐ vomiting ☐ burp ☐ flatulence
☐ temperature rise above 37°C ☐ other: ____________________
____

2.52. Do you have the diseases listed below?
○ no ○ yes, specifically:
☐ neurological diseases (write) ____________________
☐ skin diseases (write) ____________________
☐ adrenal gland disease
☐ bronchial asthma
☐ diseases of the thyroid gland (write) ____________________
☐ pituitary gland diseases
☐ type 2 diabetes
☐ hypertension (the degree of the disease) ____________________

2.53. Have you asked for psychiatric help?
○ yes
○ no

2.54. How often do you drink alcohol?
○ never
○ 1 time per month or less
○ 1-2 times a week
○ every day

2.55. Have you taken medications in the last 3 months (if so, which ones)?
○ no ○ yes, specifically:
☐ antibiotics ____________________
____
☐ cytostatics ____________________
____

☐ corticosteroids ______________________________

☐ immunosuppressants ______________________________

☐ antiviral drugs ______________________________

☐ antifungal ______________________________

☐ antiparasitic ______________________________

☐ other (write) ______________________________

☐ Do you take any medications all the time:

____

3. Consent to the processing of personal data

☐ I give my consent to the processing of the entered personal information in accordance with Federal Law №152 dated 07/27/2006 "On Personal Data".
